# Novel use of carboplatin, etoposide and durvalumab as neoadjuvant therapy in treatment of small cell bladder cancer

**DOI:** 10.1016/j.eucr.2025.103165

**Published:** 2025-08-13

**Authors:** Victoria Gonzalez, Edward Linton, Timothy Boyce, Laila Dahmoush, Helen Y. Hougen

**Affiliations:** a Department of Urology, University of Iowa Hospitals and Clinics, 200 Hawkins Dr, Iowa City, IA, 52242, USA; b Department of Ophthalmology, University of Iowa Hospitals and Clinics, 200 Hawkins Dr, Iowa City, IA, 52242, USA; c Department of Pathology, University of Iowa Hospitals and Clinics, 200 Hawkins Dr, Iowa City, IA, 52242, USA

**Keywords:** Urinary bladder neoplasms, Small cell carcinoma, Immune checkpoint inhibitor, Retinopathy

## Abstract

Small cell carcinoma of the bladder (SCCB) is a rare and aggressive variant of bladder cancer requiring multimodal treatment. We present a case of an 82-year-old male with SCCB with bulky pelvic adenopathy treated with a novel neoadjuvant combination of carboplatin, etoposide, and durvalumab with complete response of the small cell component. We also describe a rare complication of retinopathy resulting from this treatment. This study presents the potential efficacy and promise of adding immunotherapy to neoadjuvant chemotherapy for SCCB to achieve a long-term cure.

## Highlights

• Checkpoint inhibitors as neoadjuvant therapy for small cell bladder cancer (SCCB).
• Complete pathologic response in SCCB to durvalumab, cisplatin, and etoposide.
• Complication of retinopathy from neoadjuvant carboplatin, etoposide, durvalumab.

## 1 Introduction

Small cell carcinoma of the bladder (SCCB) is a rare variant of bladder cancer that comprises approximately 1% of all bladder cancers. They are aggressive entities and often present at advanced stages with a short overall survival.^1^

Due to their aggressive nature and high rate of upstaging with upfront cystectomy, patients with small cell bladder cancer often benefit from neoadjuvant therapy.^2^ Neoadjuvant therapies are often extrapolated from those for small cell lung cancer (SCLC). Current guidelines recommend chemoradiotherapy or neoadjuvant platinum-based chemotherapy (NAC) followed by local treatment with cystectomy or radiation therapy.^3^

Recently, the addition of checkpoint inhibitors such as durvalumab and atezolizumab have become standard of care for extensive SCLC.^4^^,^^5^ Studies have suggested the addition of checkpoint inhibitors (CPIs) to neoadjuvant therapy for SCCB.^6^ In this report, we describe the novel use of the combination of carboplatin, etoposide, and durvalumab in the neoadjuvant setting for SCCB. We also describe a rare complication resulting from this treatment.

## 2 Case presentation

**Fig. 1:**
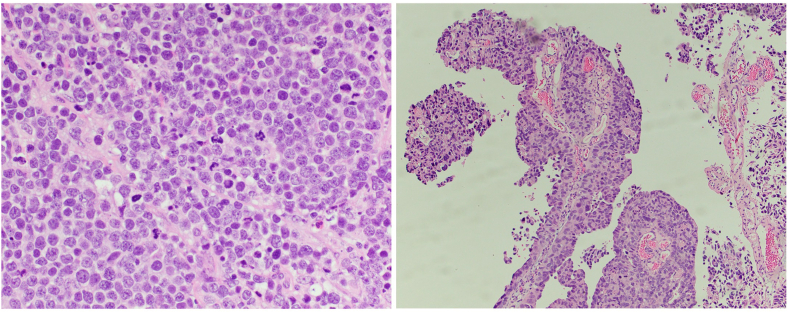
Microscopic examination of transurethral bladder tumor resection tissue prior to any treatment. Left: Evidence of small cell carcinoma of the bladder. Right: Evidence of high grade papillary urothelial cell carcinoma.

**Fig. 2:**
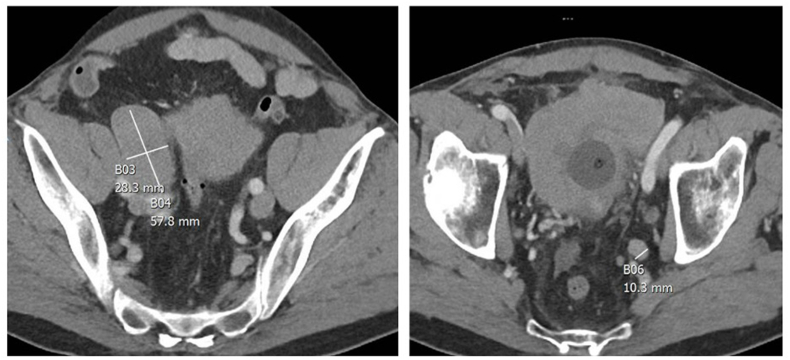
CT chest abdomen pelvis with contrast prior to systemic therapy. Left: bulky pelvic lymphadenopathy with 6cm lymph node at right external iliac. Right: Left lymphadenopathy in obturator fossa and bulky bladder disease.

An 82-year-old male with gross hematuria was found to have a 10 cm nodular bladder tumor spanning across the posterior wall to both the right and left lateral walls. A transurethral resection of the bladder tumor was performed, and pathology revealed predominantly small cell carcinoma (90%) with a minor component of conventional high grade papillary urothelial carcinoma 10% (Fig. 1). CT chest, abdomen, and pelvis (CAP) demonstrated bulky lymphadenopathy in the pelvis consistent with nodal metastasis (Fig. 2), indeterminate nodes in the retroperitoneum, but no distant metastasis. A nuclear medicine bone scan and brain MRI showed no distant metastatic disease.

A community medical oncologist initiated neoadjuvant chemotherapy and immunotherapy with carboplatin, etoposide, and durvalumab before referring the patient to our center. Staging evaluation after 3 cycles with CT CAP with contrast demonstrated significant response to neoadjuvant therapy with residual bladder thickening, marked improvement in the pelvic adenopathy, no retroperitoneal lymphadenopathy, and no evidence of distant metastasis. He completed a total of five cycles prior to surgery.

**Fig. 3:**
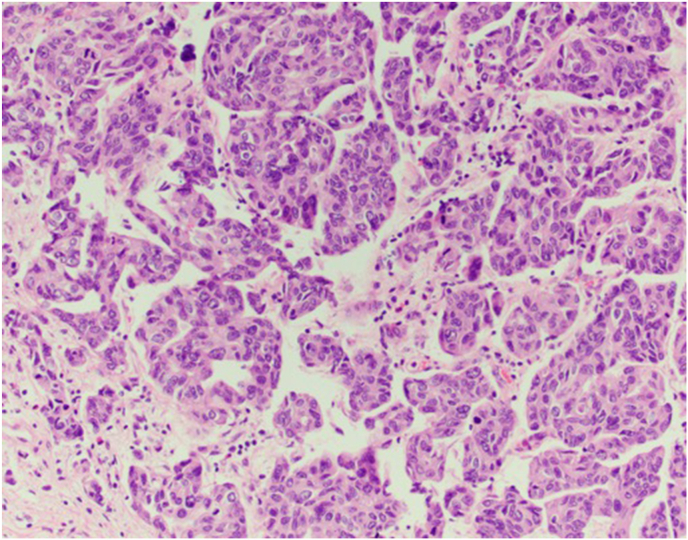
Microscopic examination of cystoprostatectomy specimen with residual micropapillary urothelial cell carcinoma after treatment with cisplatin, etoposide, and durvalumab.

He underwent robotic cystoprostatectomy with ileal conduit formation and bilateral pelvic lymph node dissection. Microscopic examination of the resected specimen showed an isolated focus of micropapillary urothelial carcinoma (Fig. 3) invading the lamina propria, with few foci of urothelial carcinoma in situ in the bladder and prostatic urethra with negative margins, ypT1N0. No residual small cell carcinoma was present. There was evidence of a strong response to neoadjuvant therapy as there was predominant fibrosis of the tumor bed with residual cancer cells occupying less than 50% of this area, tumor regression grade of 2 (TRG2).

**Fig. 4:**
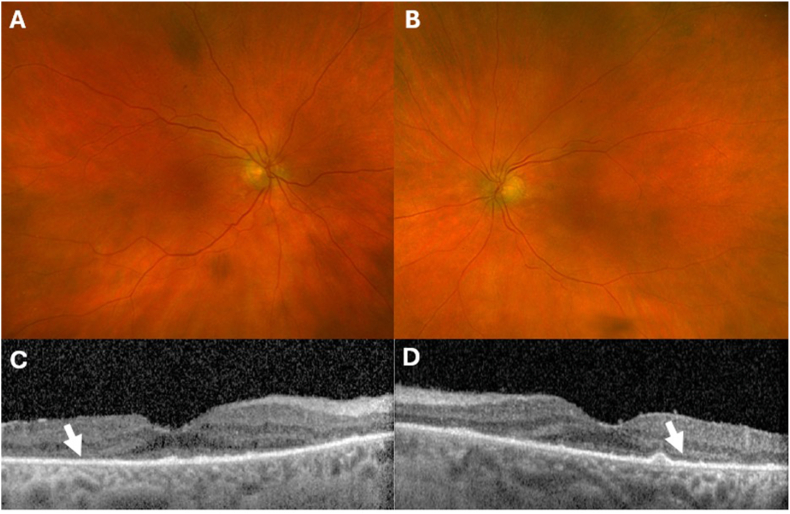
Multimodal retinal imaging. Color fundus photography (A,B) of the right eye and left eye shows unremarkable fundus appearance other than attenuated vasculature. Optical coherence tomography of both eyes (C, D) reveals diffuse outer retinal loss (white arrows).

In the months leading up to his cystectomy, roughly 4–6 months after starting neoadjuvant therapy, he suffered profound, painless, progressive vision loss to hand motions in each eye, from a baseline of 20/25-20/30 in each eye. A local eye exam and a brain MRI evaluating for CNS metastasis did not reveal a cause. During his postoperative hospitalization, ophthalmology found severe photoreceptor loss in both eyes (Fig. 4), which can be seen in paraneoplastic cancer-associated retinopathy (CAR) as well as in immunotherapy-induced retinopathy. We held a multidisciplinary discussion to weigh treatment options, including local steroid injections, systemic steroids, and plasmapheresis. Due to recent surgery, it was decided to defer high-dose systemic steroids and proceed with local steroid therapy. He underwent intravitreal dexamethasone implants in both eyes. He had remarkable improvement of his central vision to 20/50 in the right eye and 20/80 in the left eye. Therefore, long-term steroid implants were placed in both eyes for durable treatment.

Due to the adverse effect from systemic therapy and favorable pathologic response, he received no adjuvant therapy. At his 6-month follow-up after cystectomy, the patient had no evidence of recurrence.

## 3 Discussion

SCCB is a rare and aggressive variant of bladder cancer. Typically, initial diagnosis is made at advanced stages of the disease with 57% having regional lymph node involvement like this patient and the overall prognosis is unfavorable with a 5-year-survival rate of 10%–40%.^1^^,^^7^ Upfront surgery is associated with a high rate of pathologic upstaging and poor overall survival.^2^ Due to this, a multimodal treatment strategy is key to achieve long term cure. We highlight a novel neoadjuvant regimen prior to consolidative cystectomy in SCCB.

SCCB share similar histological findings to SCLC and thus we infer treatment regimens for SCCB from SCLC.^8^ Until recently, platinum-based chemotherapy was the mainstay neoadjuvant therapy.^9^ There is now data supporting incorporating the addition of CPIs to SCLC treatment; SCLC is known to have a high mutation rate which suggests that small cell cancer is immunogenic and could respond well to immune-checkpoint inhibitors. The CASPIAN and Impower133 trials demonstrated significantly longer overall survival in patients who received the addition of the CPIs (durvalumab and atezolizumab) to their treatments of extensive stage SCLC.^4^^,^^5^

There is emerging data supporting the use of CPIs as neoadjuvant therapy for SCCB. Patients with predominant variant histology bladder cancer (5% small cell) receiving neoadjuvant pembrolizumab experienced a 37% ypT0 rate.^6^ One case report of a patient with localized small cell bladder cancer who underwent upfront cystectomy and developed distant metastasis on adjuvant carboplatin and etoposide, and subsequently received carboplatin, etoposide, and atezolizumab. The patient achieved complete and sustained radiographic response at 12 months.^10^ These data support the promise of combination chemo- and immune-therapy in this patient population.

While CPIs are generally well-tolerated, immune-mediated retinopathy is a rare but devastating adverse effect. CPIs are known to cause a spectrum of immune-mediated toxicities, including ocular (uveitis, iritis, etc.) toxicities. Two potential mechanisms include off-target immune response against normal ocular tissue or paraneoplastic syndrome triggered by CPIs, and incidence range between 1 and 4%.^11^ Vision loss in CAR is caused by circulating antibodies formed against the retinal proteins in the presence of systemic cancer and it is most frequently associated with SCLC.^12^^,^^13^ Since there are similarities between SCLC and SCCB, it's plausible that CAR may occur more frequently in SCCB. Ultimately, we do not know the exact mechanism of this patient's retinopathy. Fortunately, these events are extraordinarily rare; The CASPIAN trial, which randomized patients to the addition of durvalumab to platinum-etoposide, showed similar safety profiles of the regimens between groups and no ophthalmic events were reported.^5^ This patient ultimately was treated with intravitreal steroids which led to partial visual recovery and stabilization of his disease. Nonetheless, this patient's retinopathy had a significant impact on his quality of life, due to permanent vision loss. It is possible that if the adverse event was more promptly recognized and evaluated, the patient would have benefited from early treatment and less visual disability. As CPIs are increasingly used in oncologic treatments, all members of a patient's treatment team should be aware of the various presentations of immune-mediated toxicities.

This is a first report of complete pathologic response by small cell bladder cancer to combination chemo-immunotherapy with durvalumab, cisplatin, and etoposide. This case contributes to the emerging literature of the potential efficacy and promise of adding immunotherapy to neoadjuvant chemotherapy for SCCB, a rare and aggressive entity requiring multimodal treatment to achieve long-term cure.

## 4 Conclusion

In conclusion, this report describes successful management of SCCB with neoadjuvant carboplatin, etoposide and durvalumab followed by radical cystectomy. SCCB is known to be an aggressive disease, but this treatment regimen may be an option to consider in future treatment of this disease. Further studies need to be performed to evaluate the efficacy of this. Additionally, this case describes a unique complication that could be secondary to the durvalumab, so further studies should be performed to define the safety profile of this regimen in this clinical situation.

## CRediT authorship contribution statement

**Victoria Gonzalez:** Writing – original draft, Writing – review & editing, Conceptualization. **Edward Linton:** Conceptualization, Validation, Writing – review & editing. **Timothy Boyce:** Conceptualization, Validation, Writing – review & editing. **Laila Dahmoush:** Conceptualization, Validation, Writing – review & editing. **Helen Y. Hougen:** Conceptualization, Project administration, Supervision, Validation, Writing – original draft, Writing – review & editing.

## Funding

This research did not receive any specific grant from funding agencies in the public, commercial, or not-for-profit sectors.

## Conflict of interest disclosure

The authors have no financial or non-financial interests to disclose.
